# Touch-driven advantages in reaction time but not in performance in a cross-sensory comparison of reinforcement learning

**DOI:** 10.1016/j.heliyon.2024.e41330

**Published:** 2024-12-20

**Authors:** Wenhan Sun, Isabelle Ripp, Aylin Borrmann, Maximilian Moll, Merle Fairhurst

**Affiliations:** a Faculty of Philosophy, Ludwig-Maximilians-Universität München, Germany; b Institute for Theoretical Computer Science, Mathematics and Operations Research, University of the Bundeswehr Munich, Neubiberg, Germany; c Centre for Tactile Internet with Human-in-the-Loop (CeTI), 6G Life, Technische Universität Dresden, Germany; d Acoustics and Haptics, Faculty of Electrical and Computer Engineering, Technische Universität Dresden, Germany

## Abstract

Recent research has highlighted a notable confidence bias in the haptic sense, yet its impact on learning relative to other senses remains unexplored. This online study investigated learning behaviour across visual, auditory, and haptic modalities using a probabilistic selection task on computers and mobile devices, employing dynamic and ecologically valid stimuli to enhance generalisability. We analysed reaction time as an indicator of confidence, alongside learning speed and task accuracy. Our results revealed the fastest reaction times with haptic stimuli, suggesting heightened perceptual confidence, whereas visual stimuli were the slowest, and auditory stimuli were intermediate. Despite these differences, all modalities demonstrated consistent learning speeds and accuracies. These findings support the ‘common currency’ hypothesis of perceptual confidence, facilitating modality-independent meta-representations for efficient decision-making. Additionally, reaction times were significantly faster on touch-based mobile devices compared to computers, underscoring the metacognitive efficiency of haptic feedback in technology-enhanced environments. The combination of faster reaction time in the haptic modality without sacrificing accuracy and the enhanced efficiency of touch-based interfaces advocates for the integration of haptics in technological designs to boost efficiency while maintaining a high level of precision.

## 1 Introduction

Confidence in our sensory experiences is fundamental to human cognition and behaviour, however not all senses are trusted equally. Recent research and our previous study [1] highlighted that, despite vision providing accurate evidence, people often ‘fact-check’ through touch, suggesting a greater perceptual certainty associated with tactile experiences [2,3]. This reliance on touch may stem from its ability to offer more detailed information about an object's properties [4] and provide a convincing validation of an object's presence [5,6], leading to enhanced immediacy and confidence [7,8]. For instance, we can better detect an object's texture, temperature, and shape through touch than through sight [9]. Jenkins and Lumpkin [10] characterise touch as the most intimate sense, implying that humans may have evolved to depend more on touch when visual or auditory information is ambiguous, leading to a greater innate sense of confidence in touch-based tasks [1].

While the heightened trust in the haptic sense is evident, the impact of this perceptual confidence bias in touch on decision-making and learning is unclear. Research indicates that perceptual confidence plays a pivotal role in decision-making [11] by determining crucial decision criteria [12]. Moreover our perception stems from an integrated blend of cues from different sensory modalities [13], and the significance given to each cue is influenced by its reliability. High confidence can lead to quicker, more decisive decisions, and has been shown to deeply influence decision making strategies and learning outcomes [14,15]. Integrating sensory perception into the framework of confidence-mediated decision-making provides valuable insights into decision making and learning processes. Sensory perception involves inferring the causes of uncertain sensory evidence [16], and observers can estimate the validity of their perceptual decisions by rating their confidence [17]. These metacognitive judgments reflect the observer's confidence in the quality and reliability of the information received through their senses [18], and confidence is deeply tied to the quality and reliability of information received through our senses. Bayesian theories propose that the brain encodes intricate patterns of uncertainty within its internal representations and computations, and integrate sensory evidence with prior beliefs to generate coherent interpretations of our environment [19,20]. Here sensory evidence plays an indispensable role in shaping our decisions and learning trajectories [[21], [22], [23]]. In tasks related to perceptual categorisation, humans adapt their decision threshold based on sensory ambiguity influencing decision making [1,24]. Additionally, computational psychologists have highlighted that humans not only hold estimates of perceptual confidence but also weave these levels of confidence into their decision-making [25,26]. All in all, perceptual confidence seems to significantly impact and correlate with performance [27,28] and decision making [11] across sensory modalities.

Building on our discussion of the role and trust in sensory modalities, the next essential step is to examine their implications in learning behaviour. In contrast to a predominant focus on visual research, tactile and auditory studies are only marginally represented, particularly in cross-sensory comparisons. Nonetheless, a few notable exceptions [3,29] provide some key insight. Faivre et al. [29] examined supramodal mechanisms of metacognition across auditory, visual, and tactile domains. The authors observed that metacognitive efficiency was consistent across these sensory modalities, which suggests that metacognitive processes operate via shared mechanisms. Klever et al. [3] delved into the concept of perceptual confidence serving as a “common currency” between the visual and tactile senses, and concluded that perceptual confidence functions on an abstract scale, which facilitates humans to assess the quality of their decisions across various sensory modalities. These findings resonate with the ‘common currency hypothesis' in neuroeconomics, which postulates that the brain utilises a specific valuation process when making choices between diverse options [[30], [31], [32], [33], [34]]. This hypothesis supports the notion of a universal metric within the brain, facilitating the evaluation of diverse information within a unified framework.

Our study aims to explore how sensory modalities affect learning dynamics in a reinforcement learning context. We investigate the relationship between perceptual confidence across sensory modalities using the proxy of reaction time, and its subsequent influence on learning behaviour and task performance metrics - learning speed and accuracy. Additionally, we examine whether documented overconfidence in haptic perception influences these metrics. In our previous work [1], we investigated ambiguous but comparable visual and haptic events in an illusory context. This study aims to extend into a non-illusionary scenario such as a reinforcement learning paradigm, which offers a more direct and translational understanding of how sensory modalities influence sensory-specific learning and decision-making dynamics.

In this study, stimuli were carefully chosen to reflect real-world experiences, enhancing the ecological validity of our research beyond traditional laboratory confines. This shift is essential as studies utilising highly controlled tasks often fail to capture the complexity of natural cognitive processes. Bottenhorn et al. [35] support this by demonstrating how naturalistic cognitive tasks reveal fundamental brain networks involved in real-life cognitive dynamics. Similarly, Ladouce et al. [36] argue for the critical need for realistic experimental designs, noting that traditional cognitive assessments do not accurately mimic the interaction between perception and action found in everyday settings. By employing stimuli that participants find familiar and engaging, our research not only bolsters scientific accuracy but also enhances the applicability of our findings to everyday cognitive experiences.

Our study aims to gain nuanced insight into different facets of decision making, particularly confidence. Reaction time has emerged as an influential metric in this domain. Previous studies have found a consistent negative correlation between confidence ratings and choice reaction time [37,38], which underscores the interpretation of faster reaction time as an indication of higher confidence. In the context of our study, faster reaction time thus might be interpreted as an external manifestation of a metacognitive process, wherein higher confidence levels are indicative of increased perceptual confidence.

The dual influence of sensory information available and time taken to make a decision on decision confidence is pivotal, as demonstrated by Kiani, Corthell, and Shadlen [39]. The relationship between decision time, evidence (i.e. sensory information available) and confidence is particularly relevant in reinforcement learning tasks, where the accumulation of evidence and the efficiency of decision-making are crucial for optimising outcomes. Our choice of a probabilistic selection task (PST) as the research paradigm was informed by these considerations, as we aim to dissect how variations in perceptual confidence affect learning and decision-making across visual, auditory, and haptic modalities. By examining these dynamics, our study seeks to provide a comprehensive understanding of the cognitive processes underlying confidence judgments and their impact on reinforcement learning. Our research underscores the importance of considering sensory modality effects in learning processes, which could in the long term contribute to the design of intuitive and efficient human-machine interfaces that better accommodate human sensory processing.

## 2 Methods

### 2.1 Participants

We conducted the experiments online using Gorilla, a validated online research platform 40, and acquired valid data from 122 participants (59 male, 62 female, 1 diverse; the majority were between 16 and 30 years of age, see Supplementary Table 1 for more details). ^.^Recruitment was conducted via emails sent to students and faculty members and sharing links on our faculty websites. No monetary reward was given to participants, but eligible students were given course credits. We created three different PSTs for each sensory modality, namely auditory, visual, and haptic. Participants were free to do any of the PSTs, but prohibited to repeat the task in any given sensory modality. In total, we acquired data from 191 complete PSTs (See Supplementary Table 1 for a breakdown of PSTs by device and Supplementary Table 2 for a breakdown of PSTs by sensory modalities).

**Figure 1:**
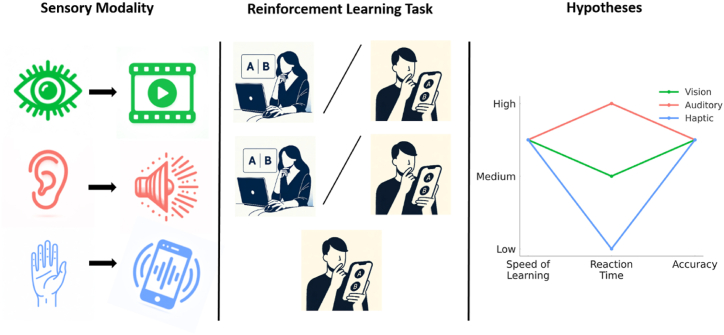
Study design and hypotheses. (left) Sensory modalities and modality specific stimuli used in the experiment, including vision and video, audition and audios, touch and haptic patterns. (middle) Reinforcement Learning Task which involves choosing between pairs of stimuli; as well as devices used in performing the task for each modality, computers and mobile devices for vision and audition, and mobile devices for touch. (right) Measures and Hypotheses with measures including speed of learning, reaction time, and accuracy. From these, we test our main hypotheses that touch would have the fastest reaction time, and that the senses are comparable in terms of speed of learning and accuracy. The icons used in this figure were created with OpenAI's DALL·E (October 2023), the graph was generated using Python (3.11.4) and the final image assembly was done in PowerPoint 2016.

All participants provided informed consent. Ethical approval was obtained from the ethics committee of the Institute of Psychology, University of the Bundeswehr Munich on April 16, 2020. No specific ethics approval number was assigned. The research was performed in accordance with the relevant guidelines and regulations of the Institute of Psychology, University of the Bundeswehr Munich, including adherence to the principles of the Declaration of Helsinki for research involving human subjects (see Fig. 1).

### 2.2 Probabilistic selection task

**Fig. 2:**
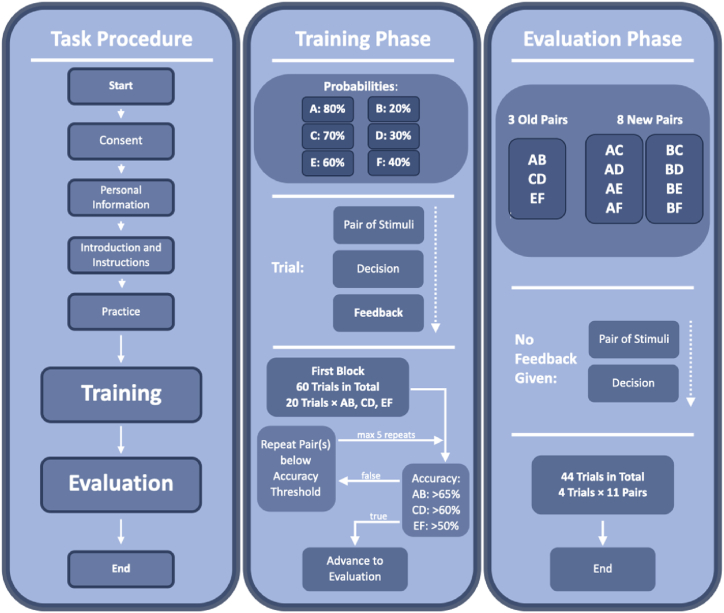
Overview of the probabilistic selection task. The left panel shows the overall procedure of the experiment detailing each step. The middle panel shows the training phase, including information on the 3 pairs of stimuli, steps within a trial, and a decision tree of the threshold. The right panel shows the evaluation phase, including pairs, steps within a trial, and a total count of trials.Figure SEQ.

We designed a PST based on Frank, Seegerber, and O'reilly [40], and Waltz et al. [41], with some modifications to optimise the task for online multi-sensory experiments. All participants conducted the PST online on a device of their choice (computers, or mobile devices such as smartphones and tablets). Participants had the flexibility to choose their preferred device and browser for the visual PST (vPST) and auditory PST (aPST). However, for the haptic PST (hPST), participation was restricted exclusively to those using Android mobile devices, to ensure uniformity in the haptic experience. The PST consisted of three phases; the practice phase, the training phase (sometimes also referred to as the acquisition phase in the literature), and the evaluation phase. See Fig. 2 for an overall task overview.

#### 2.2.1 Practice phase

**Fig. 3:**
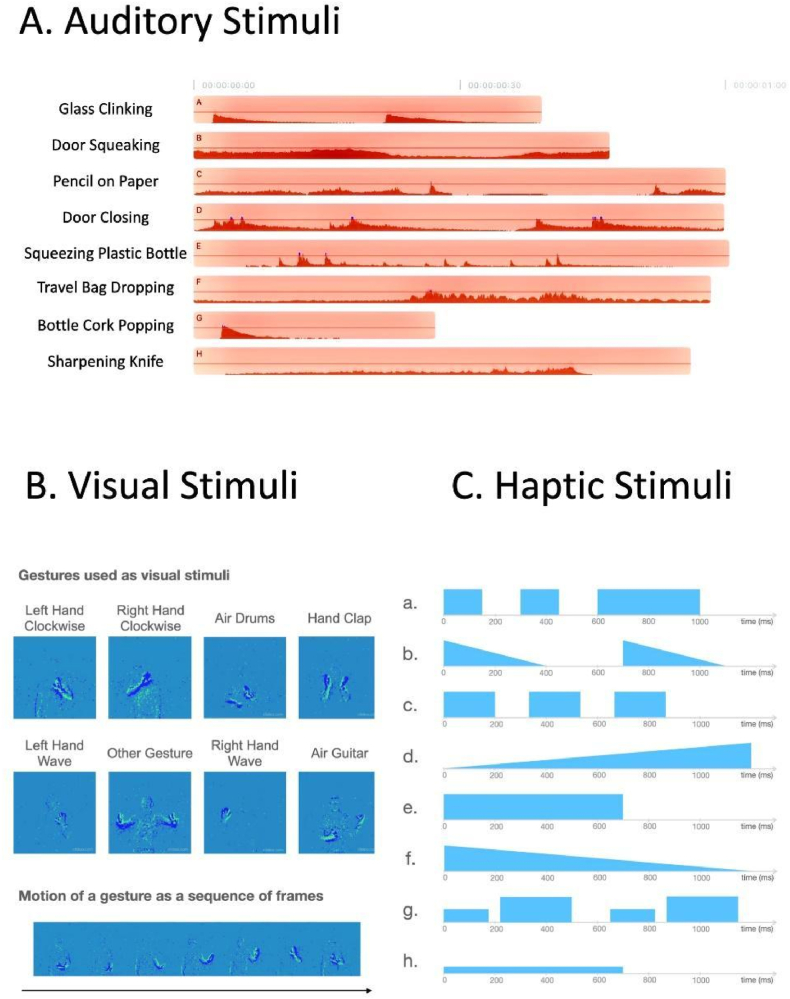
Stimuli. (A) Auditory stimuli used in aPST, waveforms demonstrate the amplitude of each sound, and x-axis demonstrate durations. (B) Visual stimuli used in vPST. One frame from each video sequence is shown to demonstrate the chosen gestures, and a sequence of frames from a gesture is used to demonstrate gesture motion. (C) Haptic stimuli used in hPST. The y-axis represents amplitude, without the distinction between sharpness and intensity. The x-axis depicts time [ms], where blue areas represent periods of active haptic acidity, and areas in blank represent periods with no haptic activity. The values depicted are approximations.

When entering the practice phase, participants were shown an introduction briefly explaining the goal of the task. Participants were presented with one pair of two sequentially-presented dynamic stimuli (see Fig. 3). The pair was presented twice, once in each order. Each time after the pair was presented, participants were instructed to choose one stimulus over the other. One stimulus of this pair was associated with implicit reward (i.e. positive feedback), while the other stimulus was associated with no reward. A choice was presented as two buttons on the screen saying “Video|Sound|Pattern 1” and “Video|Sound|Pattern 2” for vPST, aPST and hPST respectively. After choosing one stimulus over the other, feedback was given in the form of a green tick for a correct choice or a red cross for a wrong choice. On the computer, the choice was made by moving the cursor to and clicking on either of two presented buttons. On the mobile devices, the choice was made by tapping on either of the two presented buttons. An overall accuracy was shown at the end of the practice phase.

#### 2.2.2 Training phase

When entering the training phase, participants were shown an introduction. Here, the participants were provided with a brief overview of the task, including its goal and procedure. The instruction text said “In this task, you will [watch six different videos | hear six different sounds | feel six different vibration patterns]. They will be presented to you as three [video pairs | sound pairs | vibration pattern pairs]. One of the two [videos | sounds | vibration patterns] in the pair is more likely correct than the other. Your job is to figure out the overall ranking of the six [videos | sounds | vibration patterns]”.

The task in the training phase was similar to the one in the practice phase. However, subjects were now shown three different pairs (AB, CD, EF) of stimuli in random order. Both the pairs and stimuli within a pair were presented in random order. Unlike in the practice phase, different winning probabilities were associated with the different stimuli: for A: 80 %, B: 20 %; C: 70 %, D: 30 %; E: 60 %, F: 40 %. Accordingly, when pair AB is given, answer A had an 80 % probability of being correct, and B had a 20 % probability of being correct. The same rule applied to other pairs.

Through this distribution, the pairs were assigned different levels of difficulty, with AB being the easiest pair, CD the moderately difficult pair, and EF the most difficult. Participants learned through trial and error to implicitly associate higher reward probabilities with certain stimuli within each pair through the presentation of feedback after each choice.

The training phase could vary in length depending on whether repeats were needed to achieve a certain threshold. In the first block of training, 20 trials were presented for each stimulus pair, which resulted in 60 trials. After the first block of training, accuracy for each pair was calculated and compared to a threshold: AB pairs: >65 %, CD: >60 % and EF: >50 %. The thresholds were largely based on Waltz et al. 42, but were increased by one trial (5 %) to lower the chance of participants surpassing the threshold by random guessing. If the participant surpassed the accuracy threshold for all pairs, the experiment advanced to the evaluation phase. If the accuracy for one or multiple pairs were below or equal to the respective threshold, an additional training block was given, which contained 20 trials for each “unlearned” pair(s). The process repeated until the threshold was passed, and there can be a maximum of 5 repeats. After each block in the training phase, feedback was provided which lists the accuracy for each trained pair in the current block. From the training phase we collected the following variables of interest:

**Reaction Time:** the time the participants took to make a choice between two sequentially presented stimuli, measured from the presentation of the choice to the final response.

**Speed of Learning:** the total number of blocks needed for each pair in the training phase. Each participant had a minimal speed of 1 for each pair from the first block of training, and the speed for each pair increased by 1 for each repeated block. A lower number in speed reflected quicker learning and better performance.

#### 2.2.3 Evaluation phase

In the evaluation phase, participants were tested on the three old learned pairs as well as eight new pairs in random order while no feedback was given. The new pairs consisted of existing stimuli in new pairings (See Fig. 2 for more information). Different from the training phase, the correct answer in the evaluation phase was no longer probabilistic. It was determined by the selection of the stimulus with a higher winning probability compared to the paired stimulus. Hence trials were correctly answered, when a participant chose stimuli with the higher reward probability in the training phase. For example, if an old pair AB was given, A is the correct answer. If a new pair AC was given, A was the correct answer, as A was associated with a higher winning probability (80 %) than that C (70 %). Each pair was presented four times, which resulted in 44 trials. Accuracy was measured for each pair at the end of the evaluation phase. From the evaluation phase we collected the following variable of interest:

**Accuracy:** the proportion of the number of correctly answered trials over the total number of trials for each given pair in the evaluation phase. Therefore, a higher number of accuracy reflected better performance.

### 2.3 Sensory stimuli

For each sensory modality, 8 different sensory stimuli were created. Two were used only in the practice phase and 6 different sensory stimuli were randomly assigned to the 6 stimuli A, B, C, D, E, F in each PST. We aimed to maximise the comparability of stimuli across the different sensory modalities. First, we ensured that stimuli within each sensory modality were easily distinguishable from one another, preventing any potential confusion or overlap in participant responses. Second, we carefully chose stimuli that authentically represented experiences encountered in everyday life through the respective senses, enhancing ecological validity. Third, we incorporated a variety of stimuli within each modality to encompass the diverse range of sensory experiences participants might encounter. Lastly, to eliminate bias, we randomly assigned sensory stimuli to different task conditions, guaranteeing fairness and equitable task difficulty across sensory modalities. This thorough approach facilitated a direct and meaningful comparison of reaction times across different sensory channels.

#### 2.3.1 Visual probabilistic selection task

Visual stimuli were selected from and based on the DVS128 Gesture Dataset [42]. This dataset was used to build a real-time, gesture recognition system. The dataset contains 11 hand gestures from 29 subjects under 3 illumination conditions from which we selected 8 gestures, which were played for 150–200 ms. We specifically selected these dynamic visual stimuli, instead of images, to optimise comparability to stimuli from the other two naturally dynamic senses. The specific gestures we chose were: Left Hand Clockwise, Right Hand Clockwise (for the practice phase), Air Drums, Hand Clap, Left Hand Wave, Other Gesture, Right Hand Wave, and Air Guitar (for the training and evaluation phases). See Fig. 3(B) for an overview of the chosen visual stimuli, presented in the form of frames extracted from each video sequence as well as one exemplary sequence of frames. The stimuli can be accessed in our data set on Mendeley Data (see Data Availability Statement for details).

#### 2.3.2 Auditory probabilistic selection task

For our auditory stimuli, we selected ecological sounds in the form of gestural action from a sound database. After evaluating sounds based on quality and familiarity with everyday sounds, 8 sound clips were chosen, which represented sounds of glass clinking, door squeaking (for the practice phase), pencil strokes on paper, door closing, squeezing a plastic bottle, travel bag dropping on the floor, bottle cork popping, and a knife being sharpened (for the training and evaluation phases). Their lengths reflected the natural length of the sounds and ranged between 200 and 1000 ms. The stimuli can be accessed in our data set on Mendeley Data (see Data Availability Statement for details). Visualisations are provided in Fig. 3(A). Patterns A and B were used for the practice phase. Patterns C, D, E, F, G and H were used in the training and evaluation phases.

#### 2.3.3 Haptic probabilistic selection task

The haptic stimuli used in the experiment were based on Haptics.js (http://www.hapticsjs.org/), a HTML5-based online library for haptic effects in web browsers. The haptic effects were only implementable on Android devices with haptic functions and via supported browsers Chrome, Firefox, or Opera. Participants in the haptic experiment were instructed to use the above mentioned device and browsers. The haptic stimuli were created using a combination of functions provided in Haptics.js and easy-to-distinguish patterns. These stimuli varied in duration, intensity and sharpness. Examples included fade-in effects, heart beats, and consecutive quick and sharp vibrations. Their lengths were optimised for naturalness and distinguishability for each stimuli and varied between 700 and 1200 ms. Similar rhythmic haptic icons have been used before in another study [43]. The Javascript code used for haptic stimuli are listed in Supplementary Table 3, and visual approximations are provided in Fig. 3(C). Patterns A and B were used for the practice phase. Patterns C, D, E, F, G and H were used in the training and evaluation phases.

#### 2.3.4 Impact of sensory stimuli on choice reaction time

The length of each stimulus was specially set to maximise naturalness and ecological validity. For example, the lengths of the auditory stimulus of a cork popping and the haptic stimulus of 2 heart beats reflect the lengths of those sensory events in the real world. We deliberately opted not to artificially manipulate the lengths to be equal, as this would undermine how natural the stimuli felt to the participants. To assess whether differences in sensory stimuli influenced choice reaction times, we conducted comprehensive analyses, as detailed in Supplementary Table 4. We examined the effects of each specific stimulus and its order of presentation across modalities. Our results indicated minimal impact of stimulus type on reaction times, suggesting a high level of robustness in our findings. Notably, no significant differences were observed in reaction times between longer and shorter stimuli in the tested modalities.

However, an exception was observed with the sound of a knife being sharpened—an auditory stimulus of intermediate length—which resulted in a notably shorter reaction time. This anomaly appears to reflect specific characteristics of the stimulus rather than broader modality-specific sensitivities. The unique salience, potentially due to its evolutionary significance or distinctive acoustic properties, might have heightened participants’ responsiveness. This suggests that unique characteristics of stimuli, regardless of sensory modality, may influence sensory processing speeds. These effects are likely due to the emotional or cognitive impact of these stimuli, rather than being limited to their auditory nature.

### 2.4 Statistical analysis

All analyses were conducted using open-source R software version 4.2.2. (R Core Team, 2022) [44]. We conducted Linear Mixed Model (LMM) regressions with random individual effects, and investigated the effects of factors such as sensory modality, device, and pair, on our main variables of interest; reaction time, speed of learning, and accuracy. Measures from vPST were set as the default baseline, to which aPST or/and hPST measures were compared. Additional tests of estimated marginal means were performed to reveal the difference between aPST and hPST, where comparisons involve all 3 senses.

We first excluded trials where reaction time was over 1 min, and then excluded trials where reaction time was over 3 standard deviations from the mean reaction time of all participants for each sensory modality. Reaction time for remaining trials was log transformed to improve model fit. In addition, accuracy was adjusted accordingly, and overtime trials rendered incorrect post-hoc. The exclusion criteria did not affect speed.

We selected 2 stages of the training phase for analyses of reaction time. The first stage consisted of only the first block of the training (60 trials in total, 20 for each of the 3 pairs), which was equal for all participants, but may not reflect the entirety of a participant's learning process. The second stage was based on all blocks of training, which reflects the entirety of a participant's learning process, but the total number of trials included could differ for each participant depending on their number of repeated blocks.

## 3 Results

### 3.1 Reaction time

#### 3.1.1 Effect of sensory modality on reaction time

Assuming a relationship between processing time and perceptual confidence, we first investigated whether sensory modality affected reaction time without the consideration of devices. We first investigated the effect of sensory modality in the first block of training and then across the training block, to determine whether this effect is maintained.

##### 3.1.1.1 First block of training

**Fig. 4:**
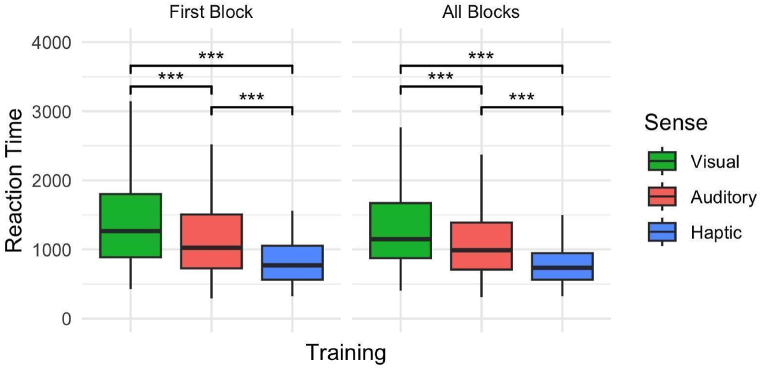
Reaction time across vPST, aPST, and hPST on all devices A) In the first block of training and B) across all training blocks. Central black bars depict the median, the boxes depict the interquartile ranges (IQR), and the whiskers depict the range at ±1.5 × IQR. Significance level depicts the results from LMM regression (Table 5, Supplementary Materials). ∗∗∗*p* < 0.001.

In the first block of training, the hPST demonstrated the fastest reaction time, followed by aPST and vPST. Specifically, compared to vPST, aPST (*β* = −0.159, *SE* = 0.030, *p* < 0.001) and hPST (*β* = −0.356, *SE* = 0.047, *p* < 0.001) have significantly lower reaction time. Notably, hPST also showed a significantly lower reaction time than aPST (*β* = −0.197, *SE* = 0.047, *p* < 0.001). Pair did not appear to have any effect on reaction time. Our model explained 72.5 % of the total variance in reaction time, of which 6.6 % could be attributed to sense and pair, and 65.9 % to individual differences. Fig. 4 depicts the corresponding box plot, and Supplementary Table 5 details the results of the regression model.

##### 3.1.1.2 All blocks of training

For all training blocks, similar trends were observed, with the hPST having the fastest reaction time, indicating that the sensory modality plays a consistent role in influencing reaction time across blocks. Specifically, compared to vPST, aPST (*β* = −0.130, *SE* = -4.388, *p* < 0.001) and hPST (*β* = −0.316, *SE* = 0.046, *p* < 0.001) showed significantly lower reaction times. A subsequent test of estimated marginal means indicated that reaction time in hPST (*β* = −0.186, *SE* = 0.046, *p* < 0.001) was also significantly lower than in aPST. Across the training block, we observed an effect of pair, with reaction time in the EF pair (*β* = 0.068, *SE* = 0.028, *p* < 0.05) being higher than in AB. Our model explained 72 % of the total variance in reaction time, of which 5.9 % could be attributed to sensory modality and pair, and 66.1 % to individual differences. Fig. 4 depicts the corresponding box plot, and Supplementary Table 5 details the results of the regression model.

#### 3.1.2 Effect of visual and auditory modality and type of device on reaction time

**Fig. 5:**
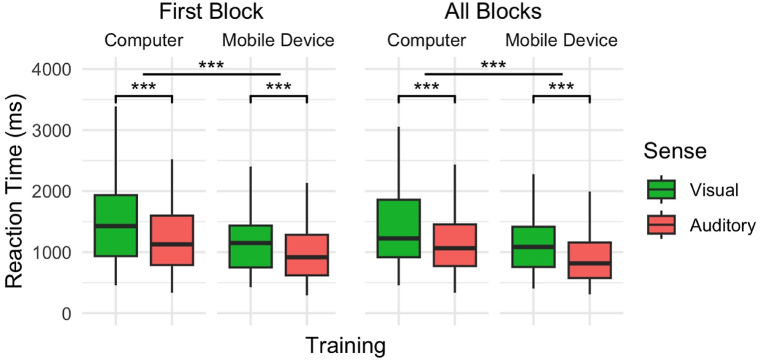
Reaction time comparison for vPST and aPST on computers and mobile devices (A) in the first training block, and (B) across all blocks of training. Central black bars depict the median, the boxes depict the interquartile ranges (IQR), and the whiskers depict the range at ±1.5 × IQR. Significance level depicts the results from LMM regression (Table 6, Supplementary Materials). ∗∗∗*p* < 0.001.

As participants were able to complete the online experiment on both computers and handheld mobile devices, we wanted to assess whether device type (computer vs mobile) had an effect on reaction time for vPST and aPST. Given that the hPST could only be done on mobile devices, we first ran LMMs investigating the effect of sensory modality and device based on vPST and aPST data, whilst excluding hPST data. The results indicate that mobile devices facilitate faster reaction time than computers for both vPST and aPST. This effect was prominent and consistent across both the first training block and all training blocks. Specifically, reaction time on mobile devices was significantly faster than on computers, and the effect appears to be strong (1st Block: *β* = −0.274, *SE* = 0.074, *p* < 0.001, All blocks: *β* = −0.277, *SE* = 0.072, *p* < 0.001). Pair did not have any effect on reaction time. Sensory modality had a strong effect on reaction time. When the factor of device was controlled, reaction time was shorter in aPST (1st Block: *β* = −0.142, *SE* = 0.030, *p* < 0.001; All blocks: *β* = −0.113, *SE* = 0.029, *p* < 0.001) than in vPST in both phases of training. Fig. 5 depicts the corresponding box plot for the first training block and for all training blocks, and Supplementary Table 6 details the results of the regression models.

Our model based on data from the first block of training explained 75.1 % of the variation in RT, of which 7.6 % is attributable to sensory modality, device, and pair; and 67.5 % to individual differences. Our model based on data from all blocks of training explained 73.2 % of the variation in RT, out of which 8 % is attributable to sensory modality, device, and pair; and 65.2 to individual differences.

#### 3.1.3 Effect of sensory modality on reaction time using mobile device

**Fig. 6:**
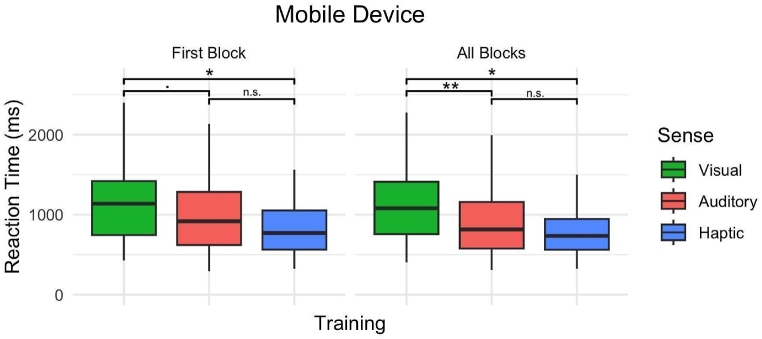
Reaction time for vPST, aPST, and hPST on mobile devices (A) in the first block of training and (B) across all training blocks. Central black bars depict the median, the boxes depict the interquartile ranges (IQR), and the whiskers depict the range at ±1.5 × IQR. Significance level depicts the results from regressions (Table 7, Supplementary Materials) and estimated marginal means. n.s.: non-significant, ∗*p* < 0.05, ∗∗*p* < 0.01, *p* < 0.10.

We further investigated the effect of all three sensory modalities on data collected from mobile devices, excluding data collected on computers and therefore excluding devices as a confounding factor. In the mobile device-only analysis, the hPST continued to demonstrate faster reaction times compared to vPST in both the first block and across all training blocks. Interestingly, the differences between aPST and hPST were not significant, implying that auditory and haptic modalities might elicit similar reaction time responses on mobile devices. Fig. 6 depicts the corresponding box plots and Supplementary Table 7 details the results of the regression models.

Specifically, aPST was marginally faster than vPST in the first block of training (*β* = −0.094, *SE* = 0.048, *p* = 0.051), and the effect was more significant in the “all blocks” model (*β* = −0.141, *SE* = 0.046, *p* < 0.01). Haptic PST was faster than vPST in both stages (1st Block: *β* = −0.141, *SE* = 0.057, *p* < 0.05, All Blocks: *β* = −0.147, *SE* = 0.055, *p* < 0.01). A subsequent test of estimated marginal means revealed that reaction time between aPST and hPST was not significantly different (1st Block: *β* = −0.047, *SE* = 0.054, *p* = 0.661, All Blocks: *β* = −0.006, *SE* = 0.052, *p* = 0.993).

Our model for the first block of training explained 77.3 % of the variance in RT, out of which 1.5 % is attributable to sense, device, and pair; and 75.8 % to individual differences. Our model for all blocks of training explained 77.8 % of the variance in RT, out of 2.6 % is attributable to sense and pair; and 75.2 % to individual differences.

### 3.2 Accuracy

#### 3.2.1 Effect of sensory modality and stimuli pair on accuracy in evaluation phase

**Fig. 7:**
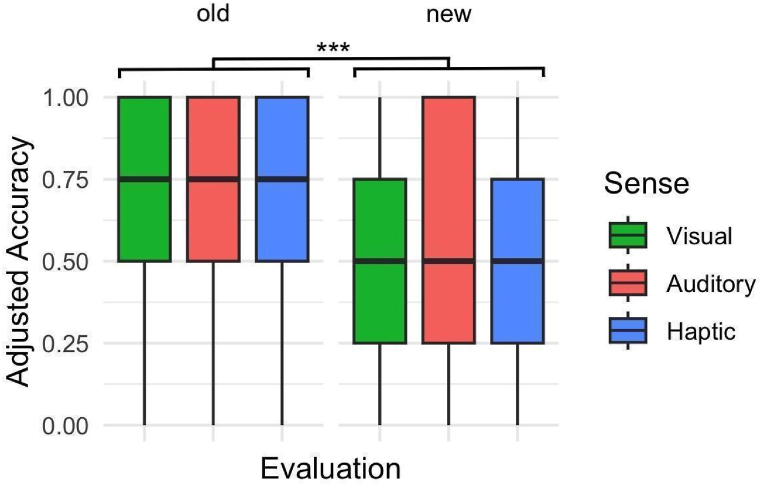
Accuracy. Box plot showing adjusted accuracy in the evaluation phase for vPST, aPST, and hPST. Central black bars depict the median, the boxes depict the interquartile ranges (IQR), and the whiskers depict the range at ±1.5 × IQR. Significance level depicts the results from regression (Table 8, Supplementary Materials) and estimated marginal means. ∗∗∗p < 0.001.

To understand whether sensory modality and pair had any effect on task performance, i.e. accuracy, in the evaluation phase, we regressed adjusted accuracy on sense and pair novelty in a LMM with random individual effects. Given that there was a large number of pairs in the evaluation phase, we created a new variable *novelty*, which distinguishes “old” pairs which appear in the training phase, and new pairs in the evaluation phase which participants have not been trained on. Fig. 7 depicts the corresponding box plot, and Supplementary Table 8 details the results of the regression model. A notable observation was that participants were more accurate in identifying “old” pairs (those seen in the training phase) compared to “new” pairs in the evaluation phase. However, the sensory modality didn't significantly impact accuracy rates. Specifically, participants achieved higher accuracy in old pairs in comparison to new ones (*β* = −0.056, *SE* = 0.016, *p* = 0.001). Adjusted accuracies in aPST (*β* = −0.007, *SE* = 0.018, *p* = 0.704), and hPST (*β* = −0.005, *SE* = 0.023, *p* = 0.837) did not appear to be significantly different from vPST, and a subsequent test of estimated marginal means indicated that adjusted accuracies of aPST and hPST were not significantly different (*β* = −0.002, *SE* = 0.023, *p* = 0.100). Overall, sense did not appear to have an effect on accuracy. Our model explained 8.8 % of the total variance in adjusted accuracy, out of which sense and novelty account for 0.6 %, and individual differences account for 8.2 %.

### 3.3 Speed of learning

#### 3.3.1 Effects of sensory modality on speed of learning in training phase

**Fig. 8:**
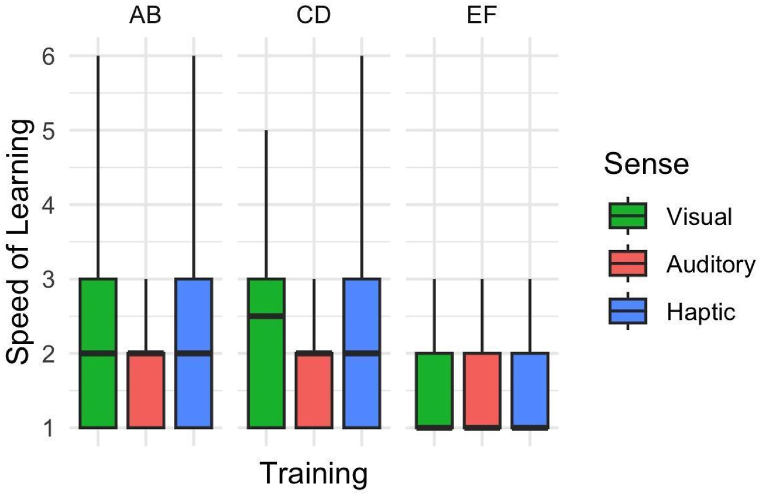
Speed of Learning. Box plots showing speed of learning in the training phase for vPST, aPST, and hPST. Central black bars depict the median, the boxes depict the interquartile ranges (IQR), and the whiskers depict the range at ±1.5 × IQR.

To investigate whether sense had an effect on the speed of learning, we regressed speed for each pair in the training phase separately on sense with LMMs with random individual effects. Fig. 8 depicts the corresponding box plot, and Supplementary Table 9 details the result of the regression models.

We found minimal differences between speeds of learning based on different senses. This suggests that while sensory modality might influence reaction time, it doesn't necessarily affect the speed at which participants learn. Specifically, speed of learning for aPST and hPST was not significantly different from vPST for pairs AB (aPST: *β* = −0.296, *SE* = 0.192, *p* = 0.124, hPST: *β* = −0.019, *SE* = 0.233, *p* = 0.935), and EF (aPST: *β* = 0.170, *SE* = 0.159, *p* = 0.287, hPST: *β* = −0.009, *SE* = 0.186, *p* = 0.961). For CD pair, aPST (*β* = −0.586, *SE* = 0.186, *p* = 0.002) did appear to have lower value (i.e faster learning), however our model failed to capture any individual effect for CD. A subsequent test of estimated marginal means indicated that speed of learning for aPST and vPST was not significantly different (AB: *β* = 0.278, *SE* = 0.236, *p* = 0.468, CD: *β* = 0.257, *SE* = 0.220, *p* = 0.472, EF: *β* = 0.179, *SE* = 0.189, *p* = 0.611). Sensory modality accounted for a small amount of variance in our models (marginal R2 for AB: = 1.2 %, CD: = 4.9 % but no random effect was captured, EF = 0.7 %).

### 3.4 Summary

Overall the tactile modality showed the fastest reaction time. Drawing on the previous discussions, this may suggest that individuals exhibit a higher level of (metacognitive) confidence when processing stimuli through the haptic modality. Faster reaction time here might be indicative of participants feeling more certain about their responses in this modality.

By contrast, the visual modality showed the slowest reaction time. Interestingly, participants' performance (learning speed and accuracy) within the visual modality was comparable to that of the tactile and auditory modalities. Slower reaction time in vPST does not necessarily mean that the information processed is less accurate, but that there might be a reduced level of internal confidence in immediate responses based on this sensory input. Although there is no comparable literature upon which a hypothesis could be formed for the auditory modality, in this exploratory investigation we observed intermediate performance in aPST, with reaction time trailing the haptic condition but surpassing visual stimuli.

Intriguingly, even after accounting for device type, the auditory modality (aPST) consistently outperformed the visual modality (vPST) in reaction time, being approximately 12–14 % faster. Furthermore, in our exhaustive three-way sensory modality comparison on only mobile devices, we observed a consistent and growing gap (i.e. throughout the training phase) in reaction time between the auditory and visual modality. This essentially replicates our computer-based findings. Despite the observed device influence on reaction time, a hierarchy in sensory modality differences remains evident throughout the analyses showing decreasing reaction time in the order of vision > auditory > haptic modality. Overall, our results show that vPST seems to be the odd one out in regards to reaction time compared to hPST and aPST, with the haptic modality being the one with the fastest reaction time.

## 4 Discussion

Our study uncovers a compelling asymmetry in reaction time across sensory modalities and task performance: Despite the clear discrepancies in reaction time, the sensory modality did not exhibit a significant influence on either learning speed or accuracy.

The notion that faster reaction time may represent a higher level of certainty or confidence when processing stimuli aligns well with the concept of confidence as understood in reinforcement learning, where confidence can help guide future choices [45,46]. Slower reaction in the visual modality may indicate lower confidence or higher uncertainty, but is not associated with worse performance. This suggests that the time taken for decision-making does not compromise the quality of the decisions made and aligns with research suggesting that confidence can operate on multiple dimensions [14]. Thus, our data suggests that different confidence levels in different sensory modalities, inferred from reaction time, do not directly impact overall task performance.

To comprehend this phenomenon, we need to dissect the multiple cognitive processes ranging from primary sensory perception to information processing and subsequent decision-making. Based on our observations, it seems that these subsequent cognitive processes might operate somewhat autonomously (in a modular manner), without significantly influencing each other. The ‘common currency hypothesis' can provide a potential explanatory framework, on why different sensory modalities, despite their differing reaction time, can yield consistent performance outcomes. This theory is supported by studies showing that confidence across different perceptual decisions can be estimated using a “common currency” and is represented in an abstract, modality-independent format [47,48]. The similarity in metacognitive performance across visual, tactile, and auditory tasks further reinforces this perspective [29,49,50]. Thus, it seems that different types of sensory information are evaluated using a unified metric, suggesting that different (subsequent) cognitive processes might function cohesively but independently. Along that line, our data and this interpretation resonate with the dual plasticity model from the field of visual perceptual learning [[51], [52], [53], [54]]. This framework argues for a task-specific selective reweighing hypothesis, suggesting that perceptual learning is underpinned by task-specific adjustments between sensory representations and decision-making pathways, rather than alterations in the sensory representations themselves (for a review see Watanabe & Sasaki [55]). We can infer from this hypothesis that the brain might prioritise specific information pathways or connections pivotal for accurate decisions, irrespective of the sensory stimulus, or its primary representation in question. Thus, our findings, when viewed through the lens of the dual plasticity model, suggest a nuanced interplay between sensory feature representations and task processing.

Cortese [56] argues for the possibility that metacognition might enhance the efficiency of reinforcement learning through the creation of simplified, “low-dimensional” meta-representations. When the brain computes confidence regarding a decision (or potentially a memory or sensory stimulus), it might transform the detailed initial representation into a more streamlined first-order meta-representation located in the prefrontal cortex. This is congruent with neural evidence highlighting domain-general metacognitive components rooted in a network encompassing the prefrontal and cingulate cortex [[57], [58], [59]]. These meta-representations exclude extraneous information, focusing on essentials, and are represented in a simpler, low-dimensional manner. When reinforcement learning utilises these simplified representations, it can process faster, avoiding the complications presented by processing more complex, high-dimensional sensory data.

In regards to our data, we propose that the differences in reaction time across sensory modalities can be primarily attributed to variations in early-stage sensory processing, as indicated by variations in perceptual confidence, as per our working definition. Perceptual confidence, as we define it in this study, refers to the efficiency with which participants respond to sensory stimuli. Variations in reaction time across sensory modalities suggest differences in early-stage sensory processing and perceptual confidence, and it's important to consider how these variations translate into higher-order cognitive processes. We argue that these sensory differences are not explicitly encoded in these suggested meta-representations that govern decision-making processes. It is likely that the meta-representations primarily incorporate higher-order factors such as choice confidence or stimulus-value relationship, rather than modality-specific differences in perceptual confidence. Two arguments support this interpretation. First, we assume that differences in perceptual confidence across the senses are not captured in these meta-representations. Second, we argue that the choice confidence possibly guided by a common currency evaluation rather than sensory and/or perceptual confidence is implemented in this meta-representation feeding into higher order cognition, such as decision making and behavioural control. An alternative to our second argument is that additional to or instead of choice confidence, stimuli values or attached value functions are implemented in the meta-representation. These meta-representations therefore could represent the relationships between discrete objects such as sensory stimuli, or more generally framed, states of the world, integrated with specific reward representations, allowing for flexible and goal-directed behaviour [[60], [61], [62], [63]]. Further research is needed to understand how such values are implemented in or recruited by such meta-representations for guiding decision making. We believe these meta-representations transcend sensory modalities, explaining the consistent learning outcomes we observed. This is consistent with findings from Masset et al. [64] which highlighted the modality-general nature of representations in the orbitofrontal cortex in rats after sensory-specific probability estimates are formed during within-sensory-modality learning.

Our finding that visual modality has the slowest reaction time stands in contrast to results obtained by Klever et al. 3 where the authors found generally that participants were quicker in responding to visual stimuli than tactile stimuli. However, in that study, “simpler” stimuli were used (i.e. two different gabor patches for the visual modality and two vibrations on both index fingers) than in the present study, making the comparability difficult. However, Klever et al. also observed a significant positive bias toward the tactile modality which again falls in line with our and previous results. Further research is needed to disentangle the relationship between nature of stimulus (e.g. complexity), sensory confidence - in terms of first order perception - and confidence in perceptual decision-making.

In light of our findings and the complexities inherent in interpreting reaction times as markers of confidence across sensory modalities, we acknowledge the foundational role of metacognitive research in informing our approach. This study aligns with existing literature that posits metacognitive evaluations—specifically, confidence judgments—as crucial yet complex processes that span across sensory experiences [3,29]. By drawing on principles from seminal works in the field, we aim to provide a nuanced understanding of how confidence, as inferred from reaction times, integrates with sensory processing and decision-making. This acknowledgement underscores our commitment to a rigorous analytical framework, informed by the broader discourse on metacognition and (sensory) perception [65].

Furthermore, we observed a significant advantage of mobile devices over computers in terms of reaction time. This could be attributed to the direct nature of interactions on mobile devices, perhaps leading to swifter motor responses. This suggests that the choice of device can have an impact on processing of the stimuli within the learning process. While we were unable to assess this aspect, it is reasonable to assume that the faster motor output underlies a likewise sped-up information processing. However, further research is needed to disentangle the effect of device, or in other words, the effect of directness, and actual time needed for information processing preceding decision making (i.e. the motor output). Overall, designing interfaces that enable direct and seamless interactions, such as touch-based interfaces, may enhance the user experience and improve task performance. The use of ecologically valid stimuli designed to mirror real-world interactions can introduce certain trade-offs that warrant discussion. The inclusion of such stimuli can lead to increased variability in sensory processing time among participants, which stems from the diverse ways individuals perceive and react to realistic scenarios. However the increased variability and potential confounds can be seen as integral elements of genuine human cognitive processes, thus providing insights into how sensory modalities function in natural settings. By capturing a broader range of human responses, our study offers a more comprehensive understanding of perceptual and cognitive dynamics as they occur in everyday life.

The use of realistic stimuli enhances the generalisability of our findings. Traditional laboratory-based studies, while offering high control over experimental variables, often fail to replicate the complexity of real-world environments where multiple sensory inputs and fluctuating conditions are the norm. By integrating stimuli that participants find familiar and engaging, we bridge the gap between experimental research and practical, everyday applications. This approach not only strengthens the external validity of our study but also increases its relevance to real-world scenarios, potentially influencing future technological designs and cognitive interventions. In analysing the potential influence of different sensory stimuli on reaction time, our findings offer robust support for the generalisability of our results across the sensory modalities tested. Notably, our comprehensive statistical analyses, as detailed in Supplementary Table 4 demonstrate that variations in stimulus type and presentation order did not significantly impact reaction time. This reinforces the reliability of our experimental design and suggests that our findings are not artefacts of specific stimuli but rather indicative of broader sensory processing dynamics.

## 5 Conclusion

In summary, our results provide valuable insights into the role of sensory stimuli in perceptual processing. The observed differences in reaction time across sensory modalities suggest that certain modalities, such as auditory and haptic, may offer more efficient perceptual processing compared to the visual modality. These findings highlight the potential for leveraging sensory modalities that allow for faster information extraction and decision-making without compromising overall accuracy. The ability of the brain to efficiently utilise high-dimensional internal representations, as suggested by previous research, may contribute to the faster perceptual processing observed in the auditory and haptic modalities. By extracting relevant information more efficiently from these modalities, individuals may experience higher confidence levels, leading to more timely decision-making. These findings have important implications for various fields, including human-computer interaction and artificial intelligence systems. Designing interfaces and interactions that capitalise on the efficiency of sensory modalities can enhance user experiences and lead to faster processing (in terms of reaction time). For example, incorporating touch-based interfaces in digital assistants or AI systems may enable more efficient and accurate interactions. Moreover, our results support the notion that perceptual learning involves a combination of sensory-specific processing and modality-independent meta-representations. While sensory-specific processing accounts for the variations in reaction time, the meta-representations that drive decision-making processes seem to prioritise relevant information regardless of the sensory modality. This suggests that the brain integrates sensory information in a modality-general manner, allowing for consistent learning performance irrespective of sensory modality.

## 6 Limitations

Several limitations need to be acknowledged in our study.

We applied a specific working definition of ‘confidence’ that is tied to reaction time. It's important to note that our notion of confidence in this context does not encompass broader concepts of confidence, such as self-assurance or certainty. Instead, we use reaction time derived confidence as a measure related to the efficiency of perceptual processing in different sensory modalities.

The measurement speed of learning (blocks of training per pair) can be unreliable at times. We based our thresholds for passing the training phase from Waltz et al. [41] with a slight increase, and expect the EF pair to be the most difficult to learn due to its lowest chance of positive feedback. However we observe that EF is learnt the fastest. Participants most likely passed the 50 % threshold for EF by random chance. In comparison, AB (accuracy threshold: 65 %) and CD (accuracy threshold: 60 %) pairs are less affected by passing by random chance, and they are also statistically analysed separately to EF. In addition, the same random chance would affect training accuracy equally for all modalities, and modality-based differences in difficulty could still manifest. As such, we believe such an imperfect measure could still provide some insight into learning performance across modalities.

## CRediT authorship contribution statement

**Wenhan Sun:** Writing – review & editing, Writing – original draft, Visualization, Validation, Software, Methodology, Investigation, Formal analysis, Data curation, Conceptualization. **Isabelle Ripp:** Writing – review & editing, Writing – original draft, Investigation, Formal analysis. **Aylin Borrmann:** Writing – original draft, Validation, Software, Methodology, Investigation, Formal analysis, Data curation, Conceptualization. **Maximilian Moll:** Writing – original draft, Supervision, Resources, Project administration, Funding acquisition, Conceptualization. **Merle Fairhurst:** Writing – review & editing, Writing – original draft, Supervision, Software, Resources, Project administration, Methodology, Investigation, Funding acquisition, Formal analysis, Data curation, Conceptualization.

## Data availability statement

Experimental data, visual stimuli, and auditory stimuli are publicly available in our dataset on Mendeley Data (https://data.mendeley.com/datasets/y9pfcs6ptx).

## Declaration of competing interest

The authors declare the following financial interests/personal relationships which may be considered as potential competing interests:Wenhan Sun reports financial support was provided by 10.13039/501100001659German Research Foundation. Merle Fairhurst reports financial support was provided by 10.13039/501100001659German Research Foundation. Merle Fairhurst reports financial support was provided by 10.13039/501100002347Federal Ministry of Education and Research of Germany. Isabelle Ripp reports financial support was provided by 10.13039/100024171Bavarian Research Institute for Digital Transformation. Aylin Borrmann reports financial support was provided by 10.13039/100024171Bavarian Research Institute for Digital Transformation. Maximilian Moll reports financial support was provided by 10.13039/100024171Bavarian Research Institute for Digital Transformation. Merle Fairhurst reports was provided by Bavarian Research Institute for Digital Transformation. If there are other authors, they declare that they have no known competing financial interests or personal relationships that could have appeared to influence the work reported in this paper.
