# Supplementary material for: Touch-driven advantages in reaction time but not in performance in a cross-sensory comparison of reinforcement learning
Source: Heliyon. 2024 Dec 20;11(1):e41330. doi: 10.1016/j.heliyon.2024.e41330 (PMC11748724; doi:10.1016/j.heliyon.2024.e41330)
Supplement: Multimedia component 1 [file mmc1.docx]

Supplementary Material

### **Table 1. Participant breakdown by age, PST breakdown by age and device.**

|  | **Participant** |  | **PST** | | |
| --- | --- | --- | --- | --- | --- |
| **Age** |  |  | **Computer** | **Phone** | Total |
| 16-20 | 20 |  | 15 | 18 | 33 |
| 21-25 | 61 |  | 59 | 37 | 96 |
| 26-30 | 27 |  | 18 | 23 | 41 |
| 31-35 | 6 |  | *0* | 8 | 8 |
| 36-40 | 2 |  | 3 | 1 | 4 |
| 40+ | 6 |  | 2 | 7 | 9 |
| Total | 122 |  | 97 | 94 | 191 |

### **Table 2. Participation distribution by PSTs across sensory modalities.**

| **Senses** | **Total** |
| --- | --- |
| Visual, Auditory, Haptic | 14 |
| Visual, Auditory | 38 |
| Visual, Haptic | 1 |
| Visual Only | 23 |
| Auditory, Haptic | 1 |
| Auditory Only | 19 |
| Haptic Only | 27 |

### **Table 3. Javascript code for haptic stimuli used in hPST**

| a | Haptics.vibrate([150,150,150,150,400]); |
| --- | --- |
| b | Haptics.fadeOut([400,300,400]) |
| c | Haptics.vibrate([200, 133, 200, 133, 200,133]); |
| d | Haptics.fadeIn(1200); |
| e | Haptics.vibrate(700); |
| f | Haptics.fadeOut(1200); |
| g | Haptics.heartbeat([500, 150, 500]); |
| h | var mediumPWM = Haptics.createPatternPWM(10, 10); mediumPWM(700); |

### **Table 4. Results from Linear Mixed Model (LMM) regressing log(reaction time) on individual sensory stimuli, and order in which a stimulus appears in a pair, with device and individual effects as random effects. Stimuli C in each modality is set as the baseline for comparison.**

| Auditory | | | | | Visual | | | | | Haptic | | | | |
| --- | --- | --- | --- | --- | --- | --- | --- | --- | --- | --- | --- | --- | --- | --- |
| log(rt) ~ order + sound + (1\|device) + (1\|participant) | | | | | log(rt) ~ order + video + (1\|device) + (1\|participant) | | | | | log(rt) ~ order + Pattern + (1\|participant) | | | | |
|  | Estimate | Std. | Pr(>\|t\|) |  |  | Estimate | Std. | Pr(>\|t\|) |  |  | Estimate | Std. | Pr(>\|t\|) |  |
| (Intercept) | 6.617 | 0.145 | 0.014 | * | (Intercept) | 6.848 | 0.122 | 0.011 | * | (Intercept) | 6.340 | 0.064 | <2e-16 | *** |
| Order - Second | 0.000 | 0.011 | 0.999 |  | Order - Second | 0.000 | 0.009 | 1.000 |  | Order - Second | 0.000 | 0.015 | 0.998 |  |
| Sound D | -0.009 | 0.019 | 0.653 |  | Video D | 0.008 | 0.016 | 0.595 |  | Pattern D | 0.019 | 0.025 | 0.451 |  |
| Sound E | 0.020 | 0.018 | 0.283 |  | Video E | 0.015 | 0.016 | 0.352 |  | Pattern E | -0.004 | 0.026 | 0.862 |  |
| Sound F | 0.007 | 0.018 | 0.711 |  | Video F | 0.020 | 0.016 | 0.207 |  | Pattern F | -0.007 | 0.026 | 0.799 |  |
| Sound G | 0.029 | 0.019 | 0.126 |  | Video G | 0.008 | 0.016 | 0.626 |  | Pattern G | 0.042 | 0.027 | 0.115 |  |
| Sound H | -0.064 | 0.019 | 0.001 | *** | Video H | 0.004 | 0.016 | 0.777 |  | Pattern H | 0.040 | 0.027 | 0.131 |  |
| --- |  |  |  |  | --- |  |  |  |  | --- |  |  |  |  |
| Conditional R2: | 0.341 |  |  |  | Conditional R2: | 0.368 |  |  |  | Conditional R2: | 0.22 |  |  |  |
| Marginal R2: | 0.001 |  |  |  | Marginal R2: | 0 |  |  |  | Marginal R2: | 0.001 |  |  |  |
| --- |  |  |  |  |  |  |  |  |  |  |  |  |  |  |
| Pairwise comparison (with the second variable as baseline) not significant except for: | | | | | Pairwise comparison with the second variable as baseline, not significant except for: | | | | | Pairwise comparison with the second variable as baseline, not significant except for: | | | | |
| H-C (β=-0.064, SE=0.019, p<0.001) | H-E (β=-0.083, SE=0.019, p<0.001) | H-F (β=-0.070, SE=0.019, p=0.003) | H-G (β=-0.092, SE=0.016, p<0.001) |  | none |  |  |  |  | none |  |  |  |  |

## **Reaction Time**

### **Table 5. Results from Linear Mixed Model (LMM) regressing log(reaction time) on sense and pair, with individual effects as random effects. vPST is set as the baseline for sense, AB for pairs.**

| First Block of Training: | | | | | Entire Training Phase: | | | | |
| --- | --- | --- | --- | --- | --- | --- | --- | --- | --- |
| LMM: log(rt) ~ sense + pair + (1\|participant) | | | | | LMM: log(rt) ~ sense + pair + speed + (1\|participant) | | | | |
|  | Estimate | Std. Error | Pr(>\|t\|) |  |  | Estimate | Std. Error | Pr(>\|t\|) |  |
| (Intercept) | 7.098 | 0.048 | < 2e-16 | *** | (Intercept) | 7.016 | 0.046 | < 2e-16 | *** |
| Sense - Auditory | -0.159 | 0.03 | 0 | *** | Sense - Auditory | -0.13 | 0.03 | 0 | *** |
| Sense - Haptic | -0.356 | 0.047 | 0 | *** | Sense - Haptic | -0.316 | 0.046 | 0 | *** |
| pair - CD | 0.014 | 0.028 | 0.629 |  | pair - CD | 0.018 | 0.028 | 0.516 |  |
| pair - EF | 0.023 | 0.028 | 0.424 |  | pair - EF | 0.068 | 0.028 | 0.013 | * |
| --- |  |  |  |  | --- |  |  |  |  |
| Conditional R2: | 0.725 |  |  |  | Conditional R2: | 0.72 |  |  |  |
| Marginal R2: | 0.066 |  |  |  | Marginal R2: | 0.059 |  |  |  |
| --- |  |  |  |  |  |  |  |  |  |
| Contrast: | Estimate | Std. Error | Pr(>\|t\|) |  | Contrast: | Estimate | Std. Error | Pr(>\|t\|) |  |
| Sense: Haptic - Auditory | -0.197 | 0.0474 | 0 | *** | Sense: Haptic - Auditory | -0.186 | 0.046 | 0 | *** |

### **Table 6. Results from Linear Mixed Model (LMM) regressing log(reaction time) on sense, device, and pair, with individual effects as random effects, and with only vPST and aPST data. vPST is set as the baseline for sense, computer for device and AB for pairs.**

| First Block of Training: | | | | | Entire Training Phase: | | | | |
| --- | --- | --- | --- | --- | --- | --- | --- | --- | --- |
| LMM: log(rt) ~ sense + device + pair + (1\|participant) | | | | | LMM: log(rt) ~ sense + device + pair + speed + (1\|participant) | | | | |
| Fixed effects: |  |  |  |  | Fixed effects: |  |  |  |  |
|  | Estimate | Std. Error | Pr(>\|t\|) |  |  | Estimate | Std. Error | Pr(>\|t\|) |  |
| (Intercept) | 7.218 | 0.058 | < 2e-16 | *** | (Intercept) | 7.146 | 0.056 | < 2e-16 | *** |
| Sense - Auditory | -0.142 | 0.030 | 0.000 | *** | Sense - Auditory | -0.113 | 0.029 | 0.000 | *** |
| device - Mobile Device | -0.274 | 0.074 | 0.000 | *** | device - Mobile Device | -0.277 | 0.072 | 0.000 | *** |
| pair - CD | 0.008 | 0.031 | 0.808 |  | pair - CD | 0.004 | 0.031 | 0.893 |  |
| pair - EF | 0.020 | 0.031 | 0.530 |  | pair - EF | 0.054 | 0.031 | 0.081 | . |
| --- |  |  |  |  | --- |  |  |  |  |
| Conditional R2: | 0.751 |  |  |  | Conditional R2: | 0.732 |  |  |  |
| Marginal R2: | 0.076 |  |  |  | Marginal R2: | 0.080 |  |  |  |

### **Table 7. Results from LMM regressing log(reaction time) on sense, and pair, with individual effects as random effect, and with only data from mobile devices. vPST is set as the baseline for sense, and AB for pairs.**

| First Block of Training: | | | | | Entire Training Phase: | | | | |
| --- | --- | --- | --- | --- | --- | --- | --- | --- | --- |
| LMM: log(rt) ~ sense + device + pair + (1\|participant) | | | | | LMM: log(rt) ~ sense + pair + speed + (1\|participant) | | | | |
|  | Estimate | Std. Error | Pr(>\|t\|) |  |  | Estimate | Std. Error | Pr(>\|t\|) |  |
| (Intercept) | 6.879 | 0.066 | <2e-16 | *** | (Intercept) | 6.815 | 0.064 | < 2e-16 | *** |
| Sense - Auditory | -0.094 | 0.048 | 0.051 | . | Sense - Auditory | -0.141 | 0.046 | 0.003 | ** |
| Sense - Haptic | -0.141 | 0.057 | 0.013 | * | Sense - Haptic | -0.147 | 0.055 | 0.008 | ** |
| pair - CD | 0.007 | 0.034 | 0.842 |  | pair - CD | 0.025 | 0.032 | 0.436 |  |
| pair - EF | 0.014 | 0.034 | 0.686 |  | pair - EF | 0.091 | 0.032 | 0.005 | ** |
| --- |  |  |  |  | --- |  |  |  |  |
| Conditional R2: | 0.773 |  |  |  | Conditional R2: | 0.778 |  |  |  |
| Marginal R2: | 0.015 |  |  |  | Marginal R2: | 0.026 |  |  |  |
| --- |  |  |  |  |  |  |  |  |  |
| Contrast: | Estimate | Std. Error | Pr(>\|t\|) |  | Contrast: | Estimate | Std. Error | Pr(>\|t\|) |  |
| Sense: Haptic - Auditory | -0.047 | 0.054 | 0.661 |  | Sense: Haptic - Auditory | -0.006 | 0.052 | 0.993 |  |

### **Table 8. Results from LMM regressing adjusted accuracy on sense and pair. vPST is set as the baseline for sense, and “old” for novelty.**

###

| LMM: adjusted accuracy ~ sense + novelty + (1\|participant) | | | | |
| --- | --- | --- | --- | --- |
|  | Estimate | Std. Error | Pr(>\|t\|) |  |
| (Intercept) | 0.63 | 0.019 | < 2e-16 | *** |
| Sense - Auditory | -0.007 | 0.018 | 0.704 |  |
| Sense - Haptic | -0.005 | 0.023 | 0.837 |  |
| Novelty - New | -0.056 | 0.016 | 0.001 | *** |
| --- |  |  |  |  |
| Conditional R2: | 0.088 |  |  |  |
| Marginal R2: | 0.006 |  |  |  |
| --- |  |  |  |  |
| Contrast: | Estimate | Std. Error | Pr(>\|t\|) |  |
| Sense: Haptic - Auditory | -0.002 | 0.023 | 0.996 |  |

###

### **Table 9. Results from LMM regressing speed for each pair on sensory modality. vPST is set as the baseline for sense.**

| LMM: Speed ~ Sense + (1 \| ID) | | | | | | | | | | | | |
| --- | --- | --- | --- | --- | --- | --- | --- | --- | --- | --- | --- | --- |
|  | AB |  |  |  | CD |  |  |  | EF |  |  |  |
|  | Estimate | Std. Error | Pr(>\|t\|) |  | Estimate | Std. Error | Pr(>\|t\|) |  | Estimate | Std. Error | Pr(>\|t\|) |  |
| (Intercept) | 2.187 | 0.144 | <2e-16 | *** | 2.421 | 0.13 | < 2e-16 | *** | 1.566 | 0.112 | <2e-16 | *** |
| Sense - Auditory | -0.296 | 0.192 | 0.125 |  | -0.585 | 0.186 | 0.002 | ** | 0.17 | 0.159 | 0.286 |  |
| Sense - Haptic | -0.018 | 0.233 | 0.937 |  | -0.328 | 0.217 | 0.132 |  | -0.008 | 0.186 | 0.964 |  |
| --- |  |  |  |  |  |  |  |  |  |  |  |  |
| Conditional R2: | 0.193 |  |  |  | NA |  |  |  | 0.037 |  |  |  |
| Marginal R2: | 0.012 |  |  |  | 0.049 |  |  |  | 0.007 |  |  |  |
| --- |  |  |  |  | --- |  |  |  | --- |  |  |  |
| Contrast: | Estimate | Std. Error | Pr(>\|t\|) |  | Contrast: | Estimate | Std. Error | Pr(>\|t\|) | Contrast: | Estimate | Std. Error | Pr(>\|t\|) |
| Sense: Auditory - Haptic | -0.278 | 0.236 | 0.468 |  | Sense: Auditory - Haptic | -0.257 | 0.220 | 0.472 | Sense: Auditory - Haptic | 0.179 | 0.189 | 0.611 |
