# Supplementary material for: Touch-driven advantages in reaction time but not in performance in a cross-sensory comparison of reinforcement learning
Source: Heliyon. 2024 Dec 20;11(1):e41330. doi: 10.1016/j.heliyon.2024.e41330 (PMC11748724; doi:10.1016/j.heliyon.2024.e41330)
Supplement: Multimedia component 2 [file mmc2.pdf]

Consent

This study is being conducted by the Institute of Psychology, Bundeswehr University (Munich, Germany).

Participation in this study is entirely voluntary and you are free to withdraw at any given moment without further consequences. All data collected and all results will only be analyzed at the group level for publications in scientific journals. Only investigators directly involved with the study will have access to the collected data, which will be treated as strictly confidential and handled in accordance with the GDPR.

To our knowledge, there are no risks involved in participating in this task.

If you have any questions regarding this study, or if you wish to raise any points or complaints before, during, or after the completion of the study, please contact us on merle.fairhurst@unibw.de.

I (please tick all):

have read the above information about the study,

have been given contact details to raise any further questions regarding the study,

understand that I can terminate and withdraw my participation in this study at any time without the need to provide a reason for doing so,

consent to investigators' use of gathered data in anonymised form for research purposes only,

understand that if I wish to withdraw this consent at a later stage, I can request the removal of my data from research datasets by contacting the investigators on merle.fairhurst@unibw.de

I agree to take part in this study. Take me to the survey.

Personal Information

Please answer the following questions before you start the experiment.

Gender: Please Select
Female
Male
Divers

Age: Please Select
16 - 20
21 - 25
26 - 30
31 - 35
36 - 40
40 +

Email address:
